# A randomized trial of ascorbic acid for the prevention of post-reperfusion syndrome during liver transplantation

**DOI:** 10.1097/HC9.0000000000000777

**Published:** 2025-07-29

**Authors:** Luis Gajate Martín, Inés de la Hoz, María Martín, Cristina Fernández, Ascensión Martín Grande, Diego Parise, Judith Villahoz, María Gómez, Mercedes Espiño, Oscar Pastor, Miguel Ángel Rodríguez Gandía, Javier Nuño

**Affiliations:** 1 Department of Anesthesiology and Critical Care, Hospital Universitario Ramón y Cajal, Madrid, Spain; 2 Department of Immunology, Hospital Universitario Ramón y Cajal, Madrid, Spain; 3 Department of Biochemistry, Hospital Universitario Ramón y Cajal, Madrid, Spain; 4 Department of Gastroenterology and Hepatology, Hospital Universitario Ramón y Cajal, Madrid, Spain; 5 Department of Liver Transplantation and Hepatology Surgery, Hospital Universitario Ramón y Cajal, Madrid, Spain

**Keywords:** acute kidney injury, cytokine response, hemodynamic instability, oxidative stress, vitamin C therapeutic use

## Abstract

**Background:**

Post-reperfusion syndrome (PRS) is a critical hemodynamic complication during liver transplantation, characterized by a significant drop in mean arterial pressure and associated with increased morbidity and mortality, systemic inflammation, and ischemia-reperfusion injury. Ascorbic acid (AA), with its antioxidant and anti-inflammatory properties, has been proposed as a potential therapeutic intervention.

**Methods:**

A single-center, double-blind, randomized controlled trial was conducted at the Hospital Universitario Ramón y Cajal, Madrid. Patients undergoing liver transplantation were randomly assigned to receive either 1500 mg of intravenous AA during the anhepatic phase (vitamin C-treated group) or 0.9% saline as a placebo (control group). The primary endpoint was PRS incidence. The secondary outcomes included inflammatory cytokine levels, postoperative renal function, and patient/graft survival.

**Results:**

Thirty-nine patients were randomized (20 controls and 19 AA-treated patients). The incidence of PRS was 30% in the control group and 10.5% in the AA group (*p*=0.235). Postoperative renal failure occurred more frequently in the AA group (68.4%) than in the control group (35%) (*p*=0.037). Four AA-treated patients (21.1%) required re-transplantation. No significant differences in cytokine levels were observed between the groups, although increases in IL-6, IL-8, and IL-10 levels were noted in patients with PRS, suggesting a stronger inflammatory response.

**Conclusions:**

AA supplementation demonstrated a noticeable trend toward reducing PRS during liver transplantation, although this was not statistically significant. An increase in renal failure and the need for re-transplantation were observed in the AA-treated group. Although the study suggests potential benefits, its small sample size limits the conclusions, pointing to the need for larger multicenter trials to determine the optimal dosage and timing.

## INTRODUCTION

Liver transplantation (LT) is the only definitive treatment for end-stage liver disease and hepatic tumors.^1^^,^^2^ The surgical procedure comprises 3 distinct phases, during which physiological instabilities may occur: (1) the hepatectomy phase, in which the native liver is dissected; (2) the anhepatic phase, when the native liver is removed, and the vascular bed for the donor liver is prepared; and (3) the neohepatic phase, during which the donor liver begins to function.^3^ The transition from the anhepatic phase to the neohepatic phase, particularly immediately after portal vein unclamping and donor liver reperfusion, is the most critical moment in LT surgery.^3^^,^^4^ One of the most serious complications that can occur during graft reperfusion is an adverse hemodynamic situation known as post-reperfusion syndrome (PRS), defined as a >30% decrease in mean arterial pressure below the baseline value within 5 minutes of reperfusion, persisting for at least 1 minute.^4^^–^^6^ PRS can be associated with severe bradycardia, decreased systemic vascular resistance, severe arrhythmias, increased pulmonary artery pressure, and even cardiac arrest. It has also been associated with acidosis, hypothermia, hyperkalemia, and hyperfibrinolysis. Moreover, patients developing PRS are at a higher risk of developing systemic inflammatory responses, post-transplant renal failure, early graft dysfunction, and death, leading to longer hospitalization times and higher associated costs.^3^^,^^6^^–^^10^

Despite improvements in surgical techniques and anesthetic management, the incidence of PRS has not decreased over the past few decades, ranging from 10% to 60% across different studies.^3^^,^^6^^–^^9^ Although various preventive measures have been tested, none have significantly affected the incidence of PRS.^3^^,^^6^^–^^9^ Furthermore, the underlying pathophysiological mechanisms and clinical factors contributing to PRS remain complex and poorly understood, making it a persistent concern for anesthesiologists.^4^^,^^8^^,^^9^ Several theories have been proposed, including the abrupt accumulation of factors upon reperfusion, such as metabolic acidosis, hyperkalemia, hypokalemia, hypothermia, air emboli, and release of vasoactive and proinflammatory mediators from both the allograft and recipient.^4^^,^^8^^,^^9^ During the reperfusion phase, many proinflammatory cytokines, such as tumor necrosis factor-alpha (TNF-α), interleukin (IL) IL-1, IL-2, and IL-8, as well as other factors, such as kallikrein and bradykinin, are released from the new liver. However, the role of each mediator in the onset and severity of PRS remains unclear.^3^^,^^4^^,^^8^^,^^9^^,^^11^

Nevertheless, there is little doubt that PRS is related to ischemia-reperfusion injury (IRI), unavoidable damage to the graft in a complex undertaking such as LT. The cellular and molecular mechanisms underlying IRI in LT are numerous.^1^ During ischemia, the interruption of oxygen and blood supply results in tissue damage, including NA^+^/K^+^-ATPase dysfunction, lactate accumulation, mitochondrial inactivation, and activation of lysozymes in hepatocytes, all of which result in the overproduction of reactive oxygen species. Both endothelial and parenchymal cells remain defenseless against the free radicals generated during ischemia at the time of reperfusion. Consequently, acute and chronic inflammatory processes are triggered by the immune system.^1^^–^^3^^,^^12^^,^^13^ After reperfusion, large amounts of proinflammatory cytokines (including IL-1β, IL-2, IL-8, and TNF-α) enter the recipient’s systemic circulation, activating the complement system and triggering inflammatory responses. This is followed by the release of additional proinflammatory mediators, such as kallikrein, bradykinin, chemokines, and activated complement, all of which are closely linked to the hemodynamic instability characteristic of PRS.^7^ The extent to which IRI contributes to PRS development and hemodynamic severity is not entirely clear,^10^^,^^14^ but it seems logical to conclude that PRS is another manifestation of IRI.

Understanding the role of oxidative stress in the pathophysiology of IRI has steered research toward the use of antioxidant supplements as prophylactic agents.^15^ Among these, ascorbic acid (AA), or vitamin C, has garnered attention because of its ability to modulate oxidative stress, inflammation, and mitochondrial function, along with its anti-apoptotic properties. In addition, AA’s favorable safety profile, low cost, and ease of administration and measurement of AA make it an attractive candidate as a potential bullet for mitigating IRI-related damage in the setting of solid organ transplantation. However, in the context of LT, the therapeutic potential of AA, including its optimal dosage, timing of administration, and synergic effects with other antioxidants, remains unclear.^2^^,^^12^ The limited number of randomized clinical trials conducted to date is highly heterogeneous in design and inconclusive in their findings.^2^ Nevertheless, preliminary preclinical studies and clinical evidence, albeit limited, indicate the potential role of AA supplementation as a therapeutic strategy in solid organ transplantation. In this regard, we previously published a protocol for a randomized controlled trial designed to assess the potential benefits of AA in reducing the incidence of PRS during LT.^16^ The present study shows and analyzes the results of this clinical trial.

## METHODS

This trial was designed in accordance with the updated CONSORT (Consolidated Standards of Reporting Trials), the Declaration of Helsinki, and the Good Clinical Practice guidelines issued by the Spanish regulatory authorities. The study was conducted at the Hospital Universitario Ramon y Cajal in Madrid and was approved by the local ethics committee. This study followed a single-center, double-blind, randomized controlled trial design.

All patients over 18 years of age who were listed for LT were eligible to participate in this study. The inclusion and exclusion criteria have been previously published and are detailed in Supplemental Table S1, http://links.lww.com/HC9/C63.^16^ Participants were randomized to receive either 1500 mg of intravenous AA (vitamin C-treated group) diluted in 100 mL of 0.9% saline or 100 mL of 0.9% saline (control group). The selected 1500 mg dose of intravenous AA was based on previous preclinical and clinical data supporting its antioxidant efficacy and safety in critically ill patients.^2^ The AA bolus was administered immediately before reperfusion, during the anhepatic phase, ~10–15 minutes prior to portal vein unclamping, as described in our previously published protocol.^16^

The primary endpoint was the proportion of patients who developed PRS following liver graft reperfusion. PRS was defined as a 30% or more decrease in mean arterial pressure within the first 5 minutes after portal-clamp release that persisted for at least 1 minute.^5^ Secondary outcomes, listed in Supplemental Table S2, http://links.lww.com/HC9/C64, included baseline and post-transplant AA levels and a range of IL profiles (measured before the intervention and 12 hours after reperfusion). Patients were monitored until day 30 post-surgery, with daily data collection through day 7, and a final follow-up visit on day 30. The severity of PRS in each patient was not formally assessed in our study (eg, in terms of vasopressor need or inotropic support beyond baseline levels), since this was beyond the scope of the original protocol.^16^ Plasma vitamin C levels were measured by HPLC with UV detection. Blood samples were collected in a 5 mL heparin tube, refrigerated on ice, centrifuged at 2500*g* to separate plasma, and immediately stored at −20°C. For plasma cytokine level measurements, blood samples were collected in 10 mL gel-dried tubes, centrifuged at 2500*g* to separate plasma, and stored at −20°C. Cytokines were measured using the high-sensitivity ELISA technique.

The current incidence of PRS at our center is ~30%. Based on the assumption of a 50% relative reduction in PRS incidence (from 30% to 15%), with an alpha risk of 5% and a power of 80%, bilateral testing, and Fleiss correction for an anticipated 10% loss rate, 268 participants (134 in each group) would be required. However, given the limited annual transplant volume at our center, this recruitment target would take 6–8 years to achieve. As such, and as previously published, this trial was designed as a pilot study with a small sample size of 35 patients per group.^16^ To attain 80% power with such a sample size, complete elimination of PRS would have been required. While such a drastic reduction was unlikely, we consider this sample to be sufficient to detect meaningful differences in the primary outcome, which would justify a future multicenter randomized controlled trial. Randomization was performed using a computer-generated sequence (PSS Statistics, SPSS Inc). To maintain blinding and ensure independence, patient enrollment and preparation for the drug study or placebo were handled by an unrelated staff member upon the patient’s arrival in the operating room.

### Statistical analysis

Quantitative variables were presented as mean, median, SD, IQR, 95% CI, and range values, as appropriate. Qualitative variables were reported as absolute and relative frequencies. The significance level for all the statistical tests was set at α=0.05. Both groups were assessed to ensure that they were balanced across all the variables. For continuous variables, the Student *t* test was applied, where normal distribution was verified. The chi-squared test was used to compare participants who experienced PRS during the intervention. Cohort proportions were compared using relative risk with 95% CIs. All statistical analyses were performed using the SPSS Statistics software.

### Ethical considerations

This study was registered in the European Union Drug Regulating Authorities Clinical Trials Database (EudraCT; 2020-000123-39) and ClinicalTrials.gov (NCT05754242). The protocol was approved by the Ethics Committee of Hospital Universitario Ramón y Cajal and the Spanish Medicines and Health Products Agency [Agencia Española del Medicamento y Productos Sanitarios (AEMPS); protocol number AEMPS-20-0052].^16^ The trial was conducted in accordance with the ethical principles governing biomedical research involving human subjects and in line with the latest version of the Declaration of Helsinki (Fortaleza, Brazil, 2013 revision), as well as applicable national regulations and AEMPS guidelines.

## RESULTS

**FIGURE 1:**
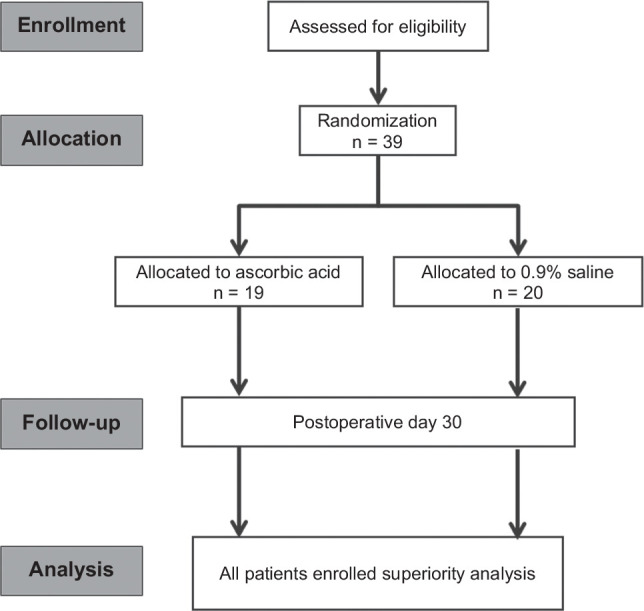
CONSORT (Consolidated Standards of Reporting Trials) flow diagram of study enrollment and randomization.

A total of 39 patients were randomized: 20 in the control group and 19 in the vitamin C-treated group. A CONSORT diagram of this study is shown in Figure 1. Donor characteristics were comparable between the groups, including demographic and laboratory variables, as shown in Supplemental Table S3, http://links.lww.com/HC9/C65. All patients received grafts from brain-dead donors, the majority of whom died from cerebrovascular accidents. Recipient characteristics were also similar across the groups, with the exception of baseline creatinine levels before transplantation, which were slightly higher in the control group. These data are presented in Supplemental Table S4, http://links.lww.com/HC9/C66.

No significant differences were observed in intraoperative variables (Table 1). All patients received grafts using the cava preservation technique (piggyback). A temporary portocaval shunt was performed in 52.6% of the patients in the vitamin C-treated group and in 40% of the patients in the control group (*p*=0.43). The duration of the anhepatic phase was nearly identical in both groups (61 min vs. 60 min in control and vitamin C-treated groups, respectively). The total operative time was slightly longer in the vitamin C-treated group; however, the difference was not statistically significant (Table 1).

**TABLE 1:** Intraoperative variables

	Control group	Vitamin C group	*p*
Crystalloids (mL, IQR)	6500 (5213–9300)	7500 (6100–9000)	0.29
Albumin (mL, IQR)	25 (0–138)	0 (0–100)	0.53
Fresh frozen plasma (mL, IQR)	0 (0–975)	0 (0–0)	0.28
Cold ischemia time (min, SD)	404.7 (92.1)	403.7 (82.1)	0.97
Median blood pressure 1 minute before unclamping (mm Hg, SD)	74.8 (15.4)	75.9 (13.6)	0.82
Colloids (mL, IQR)	0 (0–500)	0 (500–1000)	0.08
Anhepatic phase time (min, IQR)	61 (50–74)	60 (45–68)	0.52
Platelets (pool, IQR)	0 (0–1)	0 (0–2)	0.82
Hemostatic cryoprecipitate (U, IQR)	1.5 (0–4)	0 (0–4)	0.57
Biliary tract reconstruction (n)			0.32
Choledochotomy with Kehr tube	18 (90.0%)	14 (73.7%)	
Choledochotomy without Kehr tube	1 (5.0%)	4 (21.1%)	
Roux-en-Y anastomosis	1 (5.0%)	1 (5.3%)	
Temporary portocaval shunt (n)	8 (40.0%)	10 (52.6%)	0.43
Tranexamic acid (n)	6 (30.0%)	3 (15.8%)	0.45
Duration of intervention (min, SD)	374.6 (87.2)	408.3 (91.3)	0.25
Duration of anesthesia (min, SD)	477.6 (102.1)	498.6 (102.9)	0.53
Duration of anhepatic phase (min, IQR)	61 (50–74)	60 (45–68)	0.22

*Note:* IQR (p25–p75); n, number of patients.

Regarding the primary endpoint of the study, the overall incidence of PRS was 20.5% (8 patients). PRS occurred in 30.0% (6 patients) of the control group and 10.5% (2 patients) of the vitamin C-treated group. Although there was a lower incidence in the treated group, the difference was not statistically significant (*p*=0.24). The relative risk of developing PRS in the control group compared to that in the vitamin C-treated group was 2.9. The results are presented in Table 2.

**TABLE 2:** PRS incidence and relative risk

	Global	Control group	Vitamin C group	*p* ^a^
PRS patients (n)	8 (20.5%)	6 (30.0%)	2 (10.5%)	0.24
No PRS patients (n)	31	14 (70.0%)	17 (89.5%)	
Total patients (n)	39	20	19	
PRS risk (CI)		0.3 (0.145–0.519)	0.105 (0.105–0.314)	
Relative risk (CI)	2.852 (0.65–12.42) (control group vs. treated group)			0.27
OR (CI)	3.64 (0.63–20.96) (control group vs. treated group)			

*Note:* n, number of patients.

^a^ Control group versus vitamin C-treated group.

Abbreviation: PRS, post-reperfusion syndrome.

Regarding the postoperative outcomes, there were no significant differences between the groups in terms of graft dysfunction or primary failure. However, a statistically significant difference was observed in the need for re-transplantation: 4 patients in the vitamin C-treated group (21.1%) required re-transplantation compared to no patient in the control group (*p*=0.047). Table 3 presents the results. The causes of re-transplantation were as follows: combined hepatic artery and PVT in 1 patient, isolated hepatic artery thrombosis in 1 patient, unrecoverable hepatic ischemia secondary to intraoperative hemodynamic instability in another patient, and late graft dysfunction of uncertain origin in the fourth patient. One patient in the control group experienced PVT, which was successfully managed to restore normal liver function without the need for re-transplantation. One patient in the control group died of multiple organ failure secondary to sepsis 19 days after transplant.

**TABLE 3:** Postoperative variables

	Control group	Vitamin C group	*p*
Initial graft dysfunction (n)	5 (25.0%)	7 (36.8%)	0.42
Primary graft failure (n)	0	0	
**Need for re-transplantation** (n)	**0**	**4 (21.1%)**	**0.047**
Maximum ALT (U/L, IQR)	761 (424–1377)	983 (405–2321)	0.43
Maximum AST (U/L, IQR)	773 (625–2693)	1411 (653–2951)	0.46
**AKI** (n)	**7 (35.0%)**	**13 (68.4%)**	**0.037**
AKI classification			0.18
No injury (n)	13 (65.0%)	6 (31.6%)	
AKIN I (n)	1 (5.0%)	3 (15.8%)	
AKIN II (n)	1 (5.0%)	3 (15.8%)	
AKIN III (n)	5 (25.0%)	7 (36.8%)	
Transplant related sepsis (n)	4 (20.0%)	5 (26.3%)	0.72
Mechanical ventilation (n, IQR)	6 (4.3-12.7)	6 (4-45)	0.91
Reintubation (n)	1 (5.0%)	1 (5.3%)	1.0
ICU time (days, IQR)	2.7 (2.1–5.7)	4.1 (2.1–6.8)	0.56
Hospital stay (days, IQR)	14.2 (11.6–22.9)	12.7 (9.3–16.7)	0.63
Hospital readmission (n)	0 (0.0%)	1 (5.3%)	0.49
ICU readmission (n)	1 (5.0%)	1 (5.3%)	1.0
Post-transplant *Aspergillus* (n)	1 (5.0%)	2 (10.5%)	1.0
Post-transplant CMV (n)	4 (20.0%)	3 (15.8%)	1.0
Post-transplant diarrhea (n)	1 (5.0%)	2 (10.5%)	0.61
Post-transplant infections			
Surgical site infection (n)	1 (5.0%)	1 (5.3%)	1.0
Urinary tract infection (n)	3 (15.0%)	0 (0.0%)	0.23
Bacteriemia (n)	0	0	
Pneumonia (n)	3 (15.0%)	2 (10.5%)	1.0
Cholangitis (n)	2 (10.0%)	1 (5.3%)	1.0
Biliary complications			0.99
Obstruction (n)	3 (15.0%)	3 (15.8%)	
Leak (n)	3 (15.0%)	3 (15.8%)	
Neurological complications (n)	2 (10.0%)	3 (15.8%)	0.66
Portal thrombosis (n)	1 (5.0%)	2 (10.5%)	0.61
Hepatic artery thrombosis (n)	0 (0.0%)	2 (10.5%)	0.23
Reintervention (n)	5 (25.0%)	6 (31.6%)	0.65
Post-transplant hemorrhage			0.49
Digestive bleeding (n)	0 (0.0%)	1 (5.3%)	
Surgical bleeding (n)	2 (10.0%)	3 (15.8%)	
Death (n)	1 (5.0%)	0 (0.0%)	0.49

*Note:* The statistically significant variables are marked in bold. IQR (p25–p75); n, number of patients.

Abbreviations: AKI, acute kidney injury; AKIN, acute kidney injury network; CMV, cytomegalovirus; ICU, intensive care unit.

A higher incidence of acute kidney injury (AKI) was found in the vitamin C-treated group (68.4%) than in the control group (35.0%), which was statistically significant (*p*=0.04), according to the Kidney Disease: Improving Global Outcomes (KDIGO) definition. Table 3 presents the results. This difference was attributed to the higher number of patients with severe AKI. However, no significant differences were found between the groups in terms of the need for extracorporeal renal replacement therapy (Table 3). When comparing patients with and without PRS, the incidence of AKI was higher in the PRS group (62.5%) than in the non-PRS group (48.4%), although the difference was not statistically significant. The results are presented in Table 4.

**TABLE 4:** Kidney failure and graft dysfunction between patients with or without PRS

	With PRS	Without PRS	*p*
Initial graft dysfunction (n)	2 (25.0%)	10 (32.3%)	1.0
AKI KDIGO (n)	5 (62.5%)	15 (48.4%)	0.70
RRT (n)	2 (25.0%)	7 (22.6%)	1.0
AKI KDIGO classification			0.47
No injury (n)	3 (37.5%)	16 (51.6%)	
AKIN I (n)	2 (25.0%)	2 (6.5%)	
AKIN II (n)	1 (12.5%)	3 (9.7%)	
AKIN III (n)	2 (25.0%)	10 (32.3%)	

Abbreviations: AKI, acute kidney injury; AKIN, acute kidney injury network; KDIGO, Kidney Disease: Improving Global Outcomes international guidelines; n, number of patients; PRS, post-reperfusion syndrome; RRT, renal replacement therapy.

### Vitamin C levels

Pre-transplant plasma vitamin C levels were relatively low in both groups (5.10 mg/dL vs. 9.95 mg/dL in the control and treated groups, respectively). Post-transplant plasma levels significantly increased in the vitamin C-treated group, reaching 15.0 mg/dL, while they remained nearly unchanged in the control group (6.9 mg/dL). Table 5 presents the results. There were no significant differences in post-transplant vitamin C levels between the patients who developed PRS and those who did not. The values are detailed in Table 5.

**TABLE 5:** Vitamin C levels

	Control group	Vitamin C group	*p*
Pre-transplant (mg/L, IQR)	5.1 (0.6–12.8)	9.95 (3.25–15.68)	0.456
Post-transplant at 12 h (mg/L, IQR)	**6.9** (**1.2–16.3)**	**15.0** (**6.9–26)**	**0.046**
Increase	**−0.1** (**−3.9 to 3.4)**	**4.8** (**1.35–11.48)**	**0.001**
	**With PRS**	**Without PRS**	***p***
Pre-transplant (mg/L, IQR)	10.2 (3.5–19.1)	7.8 (0.9–14.68)	0.365
Post-transplant at 12 h (mg/L, IQR)	15.1 (10.2–23.1)	9.6 (1.4–19.2)	0.786
Increase	3.9 (−0.1 to 4.9)	1 (−0.6 to 6.63)	0.487

*Notes:* The statistically significant variables are marked in bold. Normal plasma vitamin C levels are 7–9 mg/L.^17^^,^^18^

IQR (p25–p75).

### Cytokine levels

No significant differences were found in the plasma cytokine levels between the groups before and after transplantation (Table 6). The only exception was IL-12, which showed a slightly higher baseline level in the control group (2.67 vs. 0.0; *p*<0.01). However, post-transplant levels were similar in both groups, as were the levels of the other cytokines analyzed.

**TABLE 6:** Pre-transplant and 12-hour post-transplant plasma interleukin levels

	Control group	Vitamin C group	*p*
Pre-transplant			
IL-1β (pg/mL)	2.39 (0.00–7.64)	2.09 (0.00–4.99)	0.61
* *TNF-α (pg/mL)	6.91 (2.36–14.99)	2.68 (0.46–8.34)	0.08
* *IL-6 (pg/mL)	24.68 (11.59–49.98)	36.35 (9.77–75.28)	0.76
* *IL-8 (pg/mL)	103.61 (40.72–128.12)	70.18 (39.46–123.38)	0.86
* *IL-10 (pg/mL)	4.175 (0.215–13.013)	2.560 (1.385–11.666)	0.96
**IL-12** (pg/mL)	**2.67** (**0.0–5.6)**	**0.0** (**0.0–0.0)**	**0.001**
Post-transplant			
IL-1β (pg/mL)	2.74 (0.25–6.08)	3.00 (0–14.25)	0.67
TNF-α (pg/mL)	4.75 (0.39–13.63)	2.96 (0.0–8.3)	0.17
IL-6 (pg/mL)	212.30 (135.25–478.86)	193.13 (100.98–454.51)	0.58
IL-8 (pg/mL)	271.16 (164.89–379.35)	282.34 (121.69–373.07)	0.76
IL-10 (pg/mL)	55.68 (28.39–214.76)	45.88 (21.01–140.52)	0.43
IL-12 (pg/mL)	0.0 (0.0–3.26)	0.0 (0.0–0.95)	0.55
Increase/difference			
IL-1β (pg/mL)	0.0 (−0.53 to 2.07)	0.0 (−1.53 to 3.16)	0.97
TNF-α (pg/mL)	−2.57 (−4.23 to 1.68)	0 (−1.89 to 0.41)	0.33
IL-6 (pg/mL)	208.32 (109.17–398.09)	200.36 (63.31–385.32)	0.65
IL-8 (pg/mL)	174.55 (78.96–367.28)	172.37 (56.07–268.29)	0.84
IL-10 (pg/mL)	52.38 (19.96–192.9)	49.44 (13.22–149.15)	0.58
IL-12 (pg/mL)	0.0 (−2.69 to 0.0)	0.0 (0.0–0.315)	0.07

*Note:* The statistically significant variables are marked in bold. Data are expressed as the median and IQR (p25–p75).

Abbreviations: IL, interleukin; TNF-α, tumor necrosis factor-alpha.

When comparing the evolution of cytokine levels between patients who developed PRS and those who did not, both groups showed similar baseline cytokine levels. However, a greater increase in IL-6, IL-8, and IL-10 levels was observed in the PRS group post-transplant, suggesting a more pronounced inflammatory response. However, the differences did not reach statistical significance. The results are presented in Table 7.

**TABLE 7:** Pre-transplant and 12-hour post-transplant IL levels between patients with or without PRS

	With PRS	Without PRS	*p*
Pre-transplant			
IL-1β (pg/mL)	3.61 (0.66–7.89)	1.95 (0–5.81)	0.32
TNF-α (pg/mL)	3.87 (2.57–10.75)	4.75 (0.78–10.34)	0.68
IL-6 (pg/mL)	31.16 (22.66–42.14)	36.35 (10.91–75.28)	0.90
IL-8 (pg/mL)	69.93 (39.05–128.79)	103.81 (60.24–117.66)	0.77
IL-10 (pg/mL)	3.91 (0.29–12.09)	4.06 (1.24–13.51)	0.74
IL-12 (pg/mL)	0.0 (0.0–2.94)	0.0 (0.0–3.83)	0.88
Post-transplant			
IL-1β (pg/mL)	4.47 (3.04–8.88)	1.95 (0–5.84)	0.09
TNF-α (pg/mL)	3.62 (0.0–5.30)	4.00 (0.0.59–8.98)	0.37
IL-6 (pg/mL)	429.945 (152.15–532.04)	210.03 (111.01–362.31)	0.18
IL-8 (pg/mL)	348.795 (277.91–586.04)	211.26 (128.41–354.87)	0.13
IL-10 (pg/mL)	109.08 (39.69–210.46)	42.93 (19.8–132.80)	0.13
IL-12 (pg/mL)	0.0 (0.0–1.43)	0.0 (0.0–2.48)	0.70
Increase/difference			
IL-1β (pg/mL)	1.29 (0.0–2.65)	0.0 (−1.22 to 1.79)	0.23
TNF-α (pg/mL)	−2.7 (−4.11 to 1.26)	−0.51 (−2.87 to 0.76)	0.46
IL-6 (pg/mL)	368.29 (114.85–489.99)	199.74 (59.39–352.72)	0.18
IL-8 (pg/mL)	256.82 (175.64–503.49)	151.22 (50.23–262.18)	0.15
IL-10 (pg/mL)	100.59 (37.68–195.51)	42.79 (10.78–126.75)	0.10
IL-12 (pg/mL)	0.0 (−0.58 to 0.0)	0.0 (−2.94 to 0.0)	0.40

*Note:* Data are expressed as median and IQR. IQR (p25–p75).

Abbreviations: IL, interleukin; PRS, post-reperfusion syndrome.

## DISCUSSION

Despite significant advancements in LT, the incidence of PRS has remained largely unchanged since its initial description by Aggarwal and colleagues in 1987.^8^^,^^9^ The underlying mechanisms of PRS are still not fully understood, hindering the development of effective preventive strategies. Hemodynamic instability associated with PRS often leads to electrolyte imbalances, such as acidosis, hypocalcemia, hyperkalemia, metabolic disturbances, and coagulopathy,^4^^,^^6^^,^^8^^,^^9^ which are likely linked to the release of inflammatory mediators with anticoagulant effects.^12^ Known risk factors associated with PRS include advanced donor and recipient age, macrovesicular steatosis, prolonged ischemia times, donor–recipient size mismatch, high MELD scores, elevated creatinine and potassium levels, low calcium levels, absence of a temporary portocaval shunt during surgery, use of the classical technique with venovenous bypass, duration of surgery, type of preservation solution, and graft flushing technique.^4^^,^^6^^,^^7^ However, despite these insights, no standardized or effective preventive measures have been established to reduce the incidence and severity of PRS.

The pathophysiology of PRS involves a cascade of inflammatory cytokines and vasodilatory agents released by both the graft and the recipient immediately after reperfusion. Increases in TNF-α, IL-1, IL-2, and IL-8 from the graft, along with kallikrein, bradykinin, chemokines, and activated complement from the recipient, have all been identified as potential mediators.^4^^,^^8^^,^^9^ PRS is generally considered to be a manifestation of IRI.^10^^,^^14^ During ischemia, enzymes such as xanthine oxidase and NADPH oxidase are activated, leading to the overproduction of reactive oxygen species and triggering oxidative stress in the graft and distant organs. This in turn activates cytokine production by KCs, which further amplifies cytokine release and proinflammatory effects.^3^^,^^7^^,^^13^ A correlation has been observed between TNF-α levels and the dosage of vasopressors required to reverse hemodynamic instability.^11^ Furthermore, poor splanchnic perfusion during the hepatectomy and anhepatic phases may compromise intestinal barrier function, leading to bacterial translocation and endotoxemia, both of which may contribute to PRS. The use of a temporary portocaval shunt during hepatectomy has been proposed as a preventive measure, although the supporting evidence remains inconclusive.^19^ Additionally, the absence of Kupffer cells during these phases impairs normal endotoxin clearance. Intestinal hypoperfusion also activates the complement system, leading to the formation of membrane attack complexes and reduction of C3 and C4 components plasma levels.^20^ Kazemi et al^21^ found that AA infusion in donors significantly reduced IL-6 mRNA and cytokine levels, with a nonsignificant trend toward reduced TN-α mRNA. However, in our study, we did not observe attenuation of IL elevation in the group treated with vitamin C compared to that in the control group. In contrast to other reports showing increased TNF-α and IL-1β,^4^^,^^9^ our study found no increase in IL-1β levels, and TNF-α levels actually decreased in the control group, with no significant changes in the vitamin C-treated group. Baseline cytokine levels were similar between the groups, with postoperative increases at 12 hours post-reperfusion in IL-6, IL-8, and IL-10 levels in patients who developed PRS, suggesting a more pronounced inflammatory response. Even if not statistically significant, this IL increase in the PSR group is important, since it points to a potential trend toward greater immune activation in patients with PRS. Further studies are needed to elucidate the role of specific cytokines in the development of PRS and assess their therapeutic potential.

Interest in high-dose AA supplementation has grown in recent years, especially in the treatment of critically ill patients with sepsis, trauma, burns, or IRI-related conditions, all of which are characterized by relative AA deficiency. The reported beneficial effects of AA supplementation in patients with sepsis and those undergoing cardiac surgery include reduced vasopressor requirements, decreased intubation time, and shorter ICU stays.^2^ AA infusion has been shown to modulate cerebral oxidative damage by reducing mitochondrial injury, preventing cell death, enhancing synaptic connections, and reducing oxidative stress markers in the brain following ischemic insult.^2^ AA appears to prevent endothelial cell damage by inhibiting NADPH oxidase activity, blocking IRF-1 transcription factor activation, and preventing excessive plasma nitric oxide (NO) production by downregulating iNOS expression.^22^ In vitro, AA has been shown to inhibit leukocyte extravasation, platelet aggregation, and apoptosis.^12^^,^^17^ However, while AA can directly scavenge reactive oxygen species, this is only possible at plasma concentrations much higher than the normal physiological levels (7–9 mg/L).^17^^,^^23^ Therefore, AA may exert most of its antioxidant effects indirectly by enhancing endogenous defenses, such as vitamin E, glutathione, or alpha-lipoic acid.^2^ AA cannot be synthesized in the human body and must be ingested daily through diet. However, enteral absorption is limited, and plasma levels cannot be raised beyond a certain threshold. Intravenous administration bypasses enterohepatic circulation, allowing for much higher plasma levels.^2^ Vitamin C is safe even at very high doses. Safety studies testing intravenous administration of AA up to 50 mg/kg reported no adverse events.^24^ Nevertheless, high enteral doses (3–5 g/day) may cause gastrointestinal discomfort and osmotic diarrhea due to a lack of absorption, while symptomatic kidney stones have been associated with intravenous AA supplementation due to calcium oxalate precipitation.^25^ However, no recent studies have reported these complications or other side effects.^2^ Our findings align with the known safety profile of AA, as no adverse events were observed.

AA supplementation in transplantation is supported by evidence of low AA levels in both donors and recipients.^2^ It has been observed that patients on the waiting list have very low antioxidant levels (including AA), likely due to chronic inflammation, inadequate dietary intake, and immunosuppressive therapies.^26^^,^^27^ Antioxidant deficiencies and low AA levels can persist for up to a year after transplantation.^2^^,^^26^^,^^27^ All these observations suggest a possible beneficial effect of intravenous AA supplementation. In this sense, reduced apoptosis of KCs has been reported in AA-treated hepatic grafts, measured via β-galactosidase activity.^28^ In agreement with other studies, we found that pre-transplant vitamin C levels were relatively low, especially in the control group (5.1 mg/L), whereas the treated group had near-normal values (9.95 mg/L).^17^^,^^23^ Although other studies have reported a postoperative decline in vitamin C,^18^ we did not observe this in our control group, possibly due to variability in timing, methodology, or patient characteristics. As expected, post-transplant levels significantly increased in the treated group, exceeding normal physiological levels.

PRS during LT has also been associated with postoperative renal dysfunction.^3^^,^^7^^,^^8^ In our study, the incidence of AKI was higher in the vitamin C-treated group (68.4%) than in the control group (35.0%) in both mild and severe forms, although the need for renal replacement therapy did not differ significantly. Intriguingly, while AA appeared to reduce the incidence of PRS, the AA group showed an increased need for re-transplantation (21.1%). This may be linked to the more severe hemodynamic disturbances observed postoperatively, or possibly due to an unknown interaction between high-dose AA and perioperative renal physiology. The exact mechanism remains uncertain and warrants further investigation in future studies. Notably, the AKI incidence was also higher in patients who developed PRS, although the difference was not statistically significant. While our study suggests a protective effect of AA against PRS, the associated increase in AKI was unexpected. However, our trial was not powered to assess AKI as a primary outcome, and the sample size was too small to draw definitive conclusions. In this sense, the LOVIT trial, which studied vitamin C in ICU patients with sepsis on vasopressors, reported higher rates of death or persistent organ dysfunction at 28 days in patients receiving a 4-day intravenous AA regimen than in those who received a placebo.^29^^,^^30^ However, other authors have argued that these results may be due to abrupt discontinuation of AA rather than the treatment itself.^31^ In our study, although not statistically significant, the vitamin C-treated group showed an unexpectedly higher incidence of AKI. An optimized trial design that includes appropriate endpoints may clarify these effects. For instance, a single high dose of AA did not reduce the incidence of delayed graft function in kidney transplants but substantially shortened its duration.^32^ Thus, larger trials are needed to optimize the timing and dosing of AA in solid organ transplantation. Additionally, our standard intraoperative management includes maintaining pre-reperfusion mean arterial pressure at a relatively low level (~75 mm Hg; Table 1) to avoid sudden hemodynamic fluctuations during reperfusion. However, we acknowledge that this strategy may have contributed to renal hypoperfusion and potentially influenced the incidence of AKI and should be re-evaluated in future studies.

The main limitation of our study is its small sample size, which limited the statistical power and precluded definitive conclusions regarding the efficacy of AA supplementation in reducing PRS in LT. Although the target sample size was 70 patients (35 per group), enrollment was limited to 39 (20 in the control group and 19 in the vitamin C-treated group) due to practical constraints in our single-center setup. We acknowledge this limitation and stress the need for multicenter trials to validate these findings with greater statistical robustness. While this limitation was foreseen,^16^ we believe that our positive results in favor of vitamin C support the feasibility and rationale for a larger trial.

In summary, although preliminary and not statistically definitive, our findings suggest the potential benefit of intravenous AA supplementation in reducing the incidence of PRS during LT (30% in the control group vs. 10.5% in the vitamin C-treated group). Owing to its antioxidant, anti-inflammatory, anti-apoptotic, and mitochondrial-stabilizing properties, along with its affordability, ease of administration, and favorable safety profile, AA represents a promising molecule for IRI protection in LT.^2^^,^^12^ Larger, multicenter clinical trials are needed to confirm its efficacy, determine the optimal dosing strategy, and evaluate whether combination therapy with other antioxidants could enhance its protective effects.

## Supplementary Material

## References

[1] Dar WA Sullivan E Bynon JS Eltzschig H Ju C . Ischaemia reperfusion injury in liver transplantation: Cellular and molecular mechanisms. Liver Int. 2019;39:788–801. 30843314 10.1111/liv.14091 PMC6483869

[2] Gori F Fumagalli J Lonati C Caccialanza R Zanella A Grasselli G . Ascorbic acid in solid organ transplantation: A literature review. Clin Nutr. 2022;41:1244–1255. 35504167 10.1016/j.clnu.2022.04.004

[3] Manning MW Kumar PA Maheshwari K Arora H . Post-reperfusion syndrome in liver transplantation—An overview. J Cardiothorac Vasc Anesth. 2020;34:501–511. 31084991 10.1053/j.jvca.2019.02.050

[4] Sahmeddini MA Tehran SG Khosravi MB Eghbal MH Asmarian N Khalili F . Risk factors of the post-reperfusion syndrome during orthotopic liver transplantation: A clinical observational study. BMC Anesthesiol. 2022;22:89. 35366808 10.1186/s12871-022-01635-3 PMC8976299

[5] Aggarwal S Kang Y Freeman JA Fortunato FL Pinsky MR . Postreperfusion syndrome: Cardiovascular collapse following hepatic reperfusion during liver transplantation. Transplant Proc. 1987;19(4 suppl 3):54–55. 3303534

[6] Umehara K Karashima Y Yoshizumi T Yamaura K . Factors associated with postreperfusion syndrome in living donor liver transplantation: A retrospective study. Anesth Analg. 2022;135:354–361. 35343925 10.1213/ANE.0000000000006002

[7] Gao Q Cai JZ Dong H . A review of the risk factors and approaches to prevention of post-reperfusion syndrome during liver transplantation. Organogenesis. 2024;20:2386730. 39097866 10.1080/15476278.2024.2386730 PMC11299628

[8] Jeong SM . Postreperfusion syndrome during liver transplantation. Korean J Anesthesiol. 2015;68:527–539. 26634075 10.4097/kjae.2015.68.6.527 PMC4667137

[9] Patrono D Romagnoli R . Postreperfusion syndrome, hyperkalemia and machine perfusion in liver transplantation. Transl Gastroenterol Hepatol. 2019;4:68. 31620650 10.21037/tgh.2019.08.12 PMC6789296

[10] Girn HRS Ahilathirunayagam S Mavor AID Homer-Vanniasinkam S . Reperfusion syndrome: Cellular mechanisms of microvascular dysfunction and potential therapeutic strategies. Vasc Endovascular Surg. 2007;41:277–293. 17704330 10.1177/1538574407304510

[11] Bezinover D Kadry Z McCullough P McQuillan PM Uemura T Welker K . Release of cytokines and hemodynamic instability during the reperfusion of a liver graft. Liver Transpl. 2011;17:324–330. 21384515 10.1002/lt.22227

[12] Kwon JH Kim D Cho H Shin BS . Ascorbic acid improves thrombotic function of platelets during living donor liver transplantation by modulating the function of the E3 ubiquitin ligases c-Cbl and Cbl-b. J Int Med Res. 2019;47:1856–1867. 30614340 10.1177/0300060518817408 PMC6567784

[13] Tsai YF Liu FC Sung WC Lin CC Chung PCH Lee WC . Ischemic reperfusion injury-induced oxidative stress and pro-inflammatory mediators in liver transplantation recipients. Transplant Proc. 2014;46:1082–1086. 24815134 10.1016/j.transproceed.2014.01.009

[14] Conti A Scala S D'Agostino P Alimenti E Morelli D Andria B . Wide gene expression profiling of ischemia-reperfusion injury in human liver transplantation. Liver Transpl. 2007;13:99–113. 17192907 10.1002/lt.20960

[15] Shi S Xue F . Current antioxidant treatments in organ transplantation. Oxid Med Cell Longev. 2016;2016:8678510. 27403232 10.1155/2016/8678510 PMC4926011

[16] Gajate L de la Hoz I Espiño M Martin Gonzalez MC Fernandez Martin C Martín-Grande A . Intravenous ascorbic acid for the prevention of postreperfusion syndrome in orthotopic liver transplantation: Protocol for a randomized controlled trial. JMIR Res Protoc. 2023;12:e50091. 38100226 10.2196/50091 PMC10757222

[17] Oudemans-van Straaten HM Man AMS de Waard MC . Vitamin C revisited. Crit Care. 2014;18:460. 25185110 10.1186/s13054-014-0460-x PMC4423646

[18] Hill A Wendt S Benstoem C Neubauer C Meybohm P Langlois P . Vitamin C to improve organ dysfunction in cardiac surgery patients—Review and pragmatic approach. Nutrients. 2018;10:974. 30060468 10.3390/nu10080974 PMC6115862

[19] Nastos C Kalimeris K Papoutsidakis N Tasoulis MK Lykoudis PM Theodoraki K . Global consequences of liver ischemia/reperfusion injury. Oxid Med Cell Longev. 2014;2014:906965. 24799983 10.1155/2014/906965 PMC3995148

[20] Bellamy MC Gedney JA Buglass H Gooi JHC Leeds Liver Group . Complement membrane attack complex and hemodynamic changes during human orthotopic liver transplantation. Liver Transpl. 2004;10:273–278. 14762866 10.1002/lt.20061

[21] Kazemi M Tabei SM Najafizadeh K Mehrabi Sisakht J Milani S Khosravi MB . Evaluation of the effect of ascorbic acid administration on gene expression level of IL-6 and TNF-α cytokines in deceased donors. Iran J Allergy Asthma Immunol. 2015;14:149–157. 25780881

[22] Wu F Tyml K Wilson JX . Ascorbate inhibits iNOS expression in endotoxin- and IFN gamma-stimulated rat skeletal muscle endothelial cells. FEBS Lett. 2002;520(1–3):122–126. 12044883 10.1016/s0014-5793(02)02804-1

[23] Hagel AF Albrecht H Dauth W Hagel W Vitali F Ganzleben I . Plasma concentrations of ascorbic acid in a cross section of the German population. J Int Med Res. 2018;46:168–174. 28760081 10.1177/0300060517714387 PMC6011295

[24] Fowler AA III Syed AA Knowlson S Sculthorpe R Farthing D DeWilde C . Phase I safety trial of intravenous ascorbic acid in patients with severe sepsis. J Transl Med. 2014;12:32. 24484547 10.1186/1479-5876-12-32 PMC3937164

[25] Thomas LDK Elinder CG Tiselius HG Wolk A Åkesson A . Ascorbic acid supplements and kidney stone incidence among men: A prospective study. JAMA Intern Med. 2013;173:386–388. 23381591 10.1001/jamainternmed.2013.2296

[26] Sotomayor CG Eisenga MF Gomes Neto AW Ozyilmaz A Gans ROB Jong WHA . Vitamin C depletion and all-cause mortality in renal transplant recipients. Nutrients. 2017;9:568. 28574431 10.3390/nu9060568 PMC5490547

[27] Lim HS Kim HC Park YH Kim SK . Evaluation of malnutrition risk after liver transplantation using the nutritional screening tools. Clin Nutr Res. 2015;4:242–249. 26566519 10.7762/cnr.2015.4.4.242 PMC4641986

[28] Ichiki A Miyazaki T Nodera M Suzuki H Yanagisawa H . Ascorbate inhibits apoptosis of Kupffer cells during warm ischemia/reperfusion injury. Hepatogastroenterology. 2008;55(82-83):338–344. 18613362

[29] Angriman F Muttalib F Lamontagne F Adhikari NKJ LOVIT Investigators . IV vitamin C in adults with sepsis: A Bayesian reanalysis of a randomized controlled trial. Crit Care Med. 2023;51:e152–e156. 37026849 10.1097/CCM.0000000000005871

[30] Lamontagne F Masse MH Menard J Sprague S Pinto R Heyland DK . Intravenous vitamin C in adults with sepsis in the intensive care unit. N Engl J Med. 2022;386:2387–2398. 35704292 10.1056/NEJMoa2200644

[31] Hemilä H Chalker E . Abrupt termination of vitamin C from ICU patients may increase mortality: Secondary analysis of the LOVIT trial. Eur J Clin Nutr. 2023;77:490–494. 36539454 10.1038/s41430-022-01254-8 PMC10115628

[32] Borran M Dashti-Khavidaki S Alamdari A Naderi N Minoo F . Evaluation of the effect of high dose intravenous vitamin C on delayed allograft function in deceased donor kidney transplantation: A preliminary report. Ren Replace Ther. 2020;6:31.

