# Supplementary material for: A randomized trial of ascorbic acid for the prevention of post-reperfusion syndrome during liver transplantation
Source: Hepatol Commun. 2025 Jul 29;9(8):e0777. doi: 10.1097/HC9.0000000000000777 (PMC12306706; doi:10.1097/HC9.0000000000000777)
Supplement: Supplementary file 1 [file hc9-9-e0777-s001.docx]

**Supplemental Digital Content Table 1. Inclusion and exclusion criteria.**

| **Inclusion criteria** | **Exclusion criteria** |
| --- | --- |
| - Aged between 18 and 67 years - Waiting for a liver transplant - Negative pregnancy test in women | - Pregnancy - Allergy to ascorbic acid - Nephrolithiasis - Glucose-6-phosphate dehydrogenase (G6PD) deficiency - Hyperoxaluria - Hyperuricemia - Haemochromatosis - Sickle cell anemia - Serum creatinine >1.2 mg/dL (women) and 1.3 mg/dL (men) - Split liver graft - Acute liver failure - Living donor liver transplantation - Grafts from donation after circulatory death - Treatment with indinavir, vitamin B12, cyclosporine, iron, deferoxamine, or disulfiram |
