# Supplementary material for: A randomized trial of ascorbic acid for the prevention of post-reperfusion syndrome during liver transplantation
Source: Hepatol Commun. 2025 Jul 29;9(8):e0777. doi: 10.1097/HC9.0000000000000777 (PMC12306706; doi:10.1097/HC9.0000000000000777)
Supplement: Supplementary file 2 [file hc9-9-e0777-s002.docx]

**Supplemental Digital Content Table 2. Lis of secondary variables**

| - The percentage of patients with primary failure and early graft dysfunction.   - Primary graft failure is defined as the complete absence of liver graft function. The patient will not survive without retransplantation. In the case of early graft dysfunction, patient survival is likely and possible without retransplantation, although morbidity will be greater than in the case of normal function. The following Olthoff criteria are used for its definition (one criterion is enough):     - Bilirubin ≥10 mg/dL on postoperative day 7.     - International normalized ratio ≥1.6 on postoperative day 7.     - Alanine aminotransferase or aspartate aminotransferase >2000 IU/mL in the first 7 postoperative days. - Length of stay in the intensive care unit and hospitalization. The number of days spent in the surgical intensive care unit and the total number of days spent in the hospital. - Dose and duration of catecholamines from the time of reperfusion. - Length of time on mechanical ventilation (hours). Time to extubation of the patient. - Vitamin C levels before transplantation and 12 hours after transplantation. Measurements are performed by high-pressure liquid chromatography with an ultraviolet detector. - Levels of inflammatory markers (IL-1β, tumor necrosis factor alpha, IL-6, IL-8, IL-10 and IL-12) before and after transplantation as measured by the high-sensitivity enzyme-linked immunosorbent assay technique. - Development of postoperative kidney failure (acute kidney injury [AKI]) during the first week. AKI is defined as an increase in serum creatinine (SCr) ≥0.3 mg/dL within 48 hours or an increase in SCr × 1.5 times baseline; known or presumed to have occurred within the previous 7 days. - Need for postoperative renal replacement therapy during the first week. Need for any type of renal replacement therapy during the first 7 days after transplantation, regardless of the technique used. - Graft and patient survival 30 days after transplantation. |
| --- |
