# Supplementary material for: A randomized trial of ascorbic acid for the prevention of post-reperfusion syndrome during liver transplantation
Source: Hepatol Commun. 2025 Jul 29;9(8):e0777. doi: 10.1097/HC9.0000000000000777 (PMC12306706; doi:10.1097/HC9.0000000000000777)
Supplement: Supplementary file 3 [file hc9-9-e0777-s003.docx]

**Supplemental Digital Content Table 3. Demographic and clinical characteristics of liver donors.**

|  | **Control Group** | **Vitamin C Group** | **p** |
| --- | --- | --- | --- |
| Age (years, SD) | 61.2 (13.2) | 63.4 (12.5) | 0.59 |
| Weight (kg, SD) | 78.6 (14.3) | 75.7 (17.1) | 0.57 |
| Height (cm, SD) | 167.9 (10.3) | 166.2 (11.9) | 0.62 |
| Sex |  |  | 0.74 |
| Female (n) | 9 (45.0%) | 9 (47.4%) |  |
| Male (n) | 11 (57.9) | 10 (52.6%) |  |
| Transfusion |  |  |  |
| FFP (U) | 0 | 0 |  |
| RBC (U) | 0 (0-0) | 0 (0-0) | 0.66 |
| Platelets (pool) | 0 | 0 |  |
| Time in ICU (h, IQR) | 72 (48-98) | 48 (24-132) | 0.44 |
| Glucose (mg/dL, SD) | 140.3 (32) | 158.9 (66.9) | 0.30 |
| Hemoglobin (g/dL, SD) | 11.9 (2.1) | 12.0 (2.3) | 0.92 |
| Aspartate aminotransferase (U/L, IQR) | 23 (16.25-48.75) | 36 (20-48.25) | 0.13 |
| Alanine aminotransferase (U/L, IQR) | 21.5 (16.25-52.25) | 29 (12-68) | 0.96 |
| Bilirubin (mg/dL, IQR) | 0.52 (0.27-0.76) | 0.59 (0.37-0.86) | 0.46 |
| CMV IgG positive (n) | 16 (80.0%) | 14 (77.8%) | 1 |
| Cardiac arrest before donation (n) | 5 (25.0%) | 4 (21.1%) | 1 |
| Dopamine (n) | 2 (10.0%) | 2 (10.5%) | 1 |
| Norepinephrine (n) | 17 (85.0%) | 14 (73.7%) | 0.38 |
| Blood group |  |  | 0.18 |
| 0 (n) | 6 (30.0%) | 10 (52.6%) |  |
| A (n) | 9 (45.0%) | 7 (36.8%) |  |
| B (n) | 5 (25.0%) | 1 (5.3%) |  |
| AB (n) | 0 (0.0%) | 1 (5.3%) |  |
| Cause of brain death |  |  | 0.73 |
| Stroke (ACVA) (n) | 15 (75.0%) | 15 (78.9%) |  |
| Traumatic brain injury (n) | 1 (5.0%) | 2 (10.5%) |  |
| Anoxia (n) | 3 (15.0%) | 1 (5.3%) |  |
| Other (n) | 1 (5.0%) | 1 (5.3%) |  |

ACVA: Acute cerebrovascular accident; CMV: Cytomegalovirus; FFP: Fres Frozen Plasma; ICU: intensive care unit; IQR: interquartile range (p25-p75); n: number of patients; RBC: Red blood cells concentrate; SD: standard deviation.
