# Supplementary material for: A randomized trial of ascorbic acid for the prevention of post-reperfusion syndrome during liver transplantation
Source: Hepatol Commun. 2025 Jul 29;9(8):e0777. doi: 10.1097/HC9.0000000000000777 (PMC12306706; doi:10.1097/HC9.0000000000000777)
Supplement: Supplementary file 4 [file hc9-9-e0777-s004.docx]

**Supplemental Digital Content Table 4**. **Demographic and clinical characteristics of liver recipients before transplantation.**

|  | **Control Group** | **Vitamin C Group** | **p** |
| --- | --- | --- | --- |
| Age (years, SD) | 52.6 (15.9) | 55.8 (14.4) | *0.52* |
| Weight (kg, SD) | 76.3 (12.1) | 79.7 (16) | *0.45* |
| Height (cm, SD) | 171.9 (7.7) | 168.5 (8.4) | *0.19* |
| Sex |  |  | *0.70* |
| Female (n) | 3 (15.0%) | 4 (21.1%) |  |
| Male (n) | 17 (85.0%) | 15 (78.9%) |  |
| Blood type |  |  | *0.49* |
| 0 (n) | 6 (30.0%) | 9 (47.4%) |  |
| A (n) | 9 (45.0%) | 8 (42.1%) |  |
| B (n) | 4 (20.0%) | 1 (5.3%) |  |
| AB (n) | 1 (5.0%) | 1 (5.3%) |  |
| Basal SBP (mmHg, SD) | 127.1 (21.9) | 139.4 (28.4) | *0.14* |
| Basal DBP (mmHg, SD) | 70.1 (11.9) | 71.7 (15.2) | *0.71* |
| Basal heart rate (bpm, SD) | 75.5 (14.9) | 79.9 (13.1) | *0.33* |
| Urea (mg/dL, SD) | 36.0 (19.6) | 30.4 (11.6) | *0.29* |
| **Creatinine** (mg/dL, SD) | **0.82 (0.15)** | **0.78 (0.2)** | ***0.038*** |
| LDH (U/L, SD) | 225.4 (80.4) | 270.1 (107.7) | *0.15* |
| Potassium (mmol/L, SD) | 4.3 (0.5) | 4.1 (0.3) | *0.10* |
| Proteins (g/dL, SD) | 6.6 (0.8) | 6.6 (0.9) | *0.84* |
| Albumin (g/dL, SD) | 3.3 (0.7) | 3.0 (0.7) | *0.24* |
| Aspartate aminotransferase (U/L, IQR) | 53 (38.3-129.3) | 44 (30-80) | *0.11* |
| Alanine aminotransferase (U/L, IQR) | 41 (27.5-70.5) | 31 (20-45) | *0.22* |
| Bilirubin (mg/dL, RV) | 4.57 (0.95-10.18) | 1.88 (0.6-5.16) | *0.20* |
| Gamma-glutamyl transferase (U/L, IQR) | 82 (46.5-214.5) | 89 (38.0-133.0) | *0.60* |
| Alkaline phosphatase (U/L, IQR) | 182 (109.3-313.0) | 121 (103.0-207.0) | *0.38* |
| Cause of liver transplant |  |  | *0.58* |
| Cirrhosis (n) | 10 (50.0%) | 18 (94.7%) |  |
| Tumor (n) | 9 (45.0%) | 0 (0.0%) |  |
| Other cause (n) | 1 (5.0%) | 1 (5.3%) |  |
| Child-Pugh score |  |  | *0.66* |
| A (n) | 8 (40.0%) | 6 (31.6%) |  |
| B (n) | 3 (15.0%) | 5 (26.3%) |  |
| C (n) | 9 (45.0%) | 8 (42.1%) |  |
| Liver disease complications |  |  |  |
| BP (n) | 2 (10.0%) | 2 (10.5%) | *1* |
| UGIB (n) | 4 (20.0%) | 6 (31.6%) | *0.48* |
| Hepatic encephalopathy (n) | 6 (30.0%) | 9 (47.4%) | *0.27* |
| Ascites (n) | 9 (45.0%) | 11 (57.9%) | *0.42* |
| Hepatopulmonary syndrome (n) | 0 (0%) | 1 (5.3%) | *0.49* |
| Hepatorenal syndrome (n) | 0 (0%) | 1 (5.3%) | *0.49* |
| Pre-transplant medical care |  |  | *0.23* |
| Home care (n) | 18 (90.0%) | 18 (94.7%) |  |
| Ward hospitalization (n) | 2 (10.0%) | 0 (0.0%) |  |
| ICU hospitalization (n) | 0 (0.0%) | 1 (5.3%) |  |
| Smoker (n) | 6 (30.0%) | 5 (26.3%) | *0.80* |
| Hypertension (n) | 3 (15.0%) | 6 (31.6%) | *0.27* |
| Diabetes Mellitus) |  |  | *0.70* |
| Insulin treatment (n) | 4 (20.0%) | 6 (31.6%) |  |
| Oral hypoglycemic agents (n) | 3 (15.0%) | 2 (10.5%) |  |
| Prothrombin time (sec, SD) | 16.2 (3.5) | 14.9 (2.3) | *0.20* |
| PTA (%, SD) | 62 (22.6) | 68.6 (21.7) | *0.36* |
| INR (SD) | 1.43 (0.35) | 1.29 (0.22) | *0.14* |
| Hemoglobin (g/dL, SD) | 12.2 (2.3) | 12.5 (2.0) | *0.64* |
| Hematocrit (%, SD) | 36.4 (6.4) | 37.1 (6.3) | *0.73* |
| Platelets (10^3^/μL, IQR) | 75.25 (54.0-134.0) | 75.1 (42.2-107.0) | *0.57* |
| Leukocytes (10^3^/μL, SD) | 5.4 (1.9) | 5.4 (2.2) | *0.99* |
| Epstein-Barr virus positive (n) | 15 (75.0%) | 15 (78.94%) | *1.0* |
| HIV positive (n) | 1 (5.0%) | 0 (0.0%) | *1.0* |
| Cytomegalovirus positive (n) | 13 (65.0%) | 17 (89.5%) | *0.23* |
| Hepatitis D virus positive (n) | 1 (5.0%) | 1 (5.3%) | *1.0* |
| Hepatitis C virus positive (n) | 4 (20.0%) | 5 (26.3%) | *0.72* |
| Hepatitis B virus positive (n) | 1 (5.0%) | 1 (5.3%) | *1.0* |
| Pre-transplant diuretic treatment (n) | 10 (5.00%) | 13 (68.4%) | *0.24* |
| Previous abdominal surgery (n) | 9 (45.0%) | 7 (36.8%) | *0.61* |

The statistically significant variables are marked in bold.

BP: spontaneous bacterial peritonitis; bpm: beats per minute; DBP: diastolic blood pressure; INR: international normalized ratio; IQR: interquartile range (p25-p75); LDH: lactate dehydrogenase; n: number of patients; PTA: prothrombin time activity; SBP: systolic blood pressure; SD: standard deviation; UGIB: upper gastrointestinal bleeding.
